# A Healthy Fear of the Unknown: Perspectives on the Interpretation of Parameter Fits from Computational Models in Neuroscience

**DOI:** 10.1371/journal.pcbi.1003015

**Published:** 2013-04-04

**Authors:** Matthew R. Nassar, Joshua I. Gold

**Affiliations:** Department of Neuroscience, University of Pennsylvania, Philadelphia, Pennsylvania, United States of America; Indiana University, United States of America

## Abstract

Fitting models to behavior is commonly used to infer the latent computational factors responsible for generating behavior. However, the complexity of many behaviors can handicap the interpretation of such models. Here we provide perspectives on problems that can arise when interpreting parameter fits from models that provide incomplete descriptions of behavior. We illustrate these problems by fitting commonly used and neurophysiologically motivated reinforcement-learning models to simulated behavioral data sets from learning tasks. These model fits can pass a host of standard goodness-of-fit tests and other model-selection diagnostics even when the models do not provide a complete description of the behavioral data. We show that such incomplete models can be misleading by yielding biased estimates of the parameters explicitly included in the models. This problem is particularly pernicious when the neglected factors are unknown and therefore not easily identified by model comparisons and similar methods. An obvious conclusion is that a parsimonious description of behavioral data does not necessarily imply an accurate description of the underlying computations. Moreover, general goodness-of-fit measures are not a strong basis to support claims that a particular model can provide a generalized understanding of the computations that govern behavior. To help overcome these challenges, we advocate the design of tasks that provide direct reports of the computational variables of interest. Such direct reports complement model-fitting approaches by providing a more complete, albeit possibly more task-specific, representation of the factors that drive behavior. Computational models then provide a means to connect such task-specific results to a more general algorithmic understanding of the brain.

The use of models to infer the neural computations that underlie behavior is becoming increasingly common in neuroscience research, especially for cognitive and perceptual tasks involving decision making and learning. As their sophistication and usefulness expand, these models become increasingly central to the design, analysis, and interpretation of experiments. We consider this development to be generally positive but provide here some perspectives on the challenges inherent to this approach, particularly when behavior might be driven by unexpected factors that can complicate the interpretation of model fits. Our goal is to raise awareness of these issues and present complementary approaches that can help ensure that our understanding of the brain does not become overly conditioned to the quality of existing models fit to particular data sets.

We illustrate these challenges using a set of models that describe the ongoing process of learning values to guide actions and that are used extensively in the field of cognitive neuroscience [1]–[13]. These models adjust expectations about future outcomes according to the difference between actual and predicted outcomes, known as the prediction error. Originally developed in parallel in both animal- and machine-learning fields [14]–[16], this relatively simple form of reinforcement-learning algorithm (often referred to as a “delta rule” because the prediction error is typically represented by the Greek symbol delta (*∂*) in the equations) has: 1) provided efficient solutions to a broad array of biologically relevant problems [15]; 2) accounted for many, but not all, learning phenomena exhibited by both human and nonhuman subjects [17], [18]; 3) provided a generative architecture that has been used to predict behavior across tasks, compare brain activity to learning variables within a single task, and explore the range of possible behaviors that one might expect to find in a variable population [19], [20]; and 4) guided an understanding of the neural computations expressed by the brainstem dopaminergic system [21]. These successes have led to the proposal that the interpretation of delta-rule model parameters fit to behavioral data from human subjects performing simple learning tasks might serve as a more precise diagnostic tool for certain mental disorders than existing methods [22]–[24]. Thus reinforcement-learning models are becoming highly influential in guiding and filtering our understanding of normal and pathological brain function.

Here we focus on the interpretation of a term in most delta-rule models called the learning rate. The learning rate, α, determines the amount of influence that the prediction error, δ, associated with a given outcome has on the new expectation of future outcomes, E:
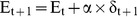
(EQ 1)As its name implies, the learning rate determines how quickly the model adapts to errors. A fixed value near zero implies that expectations are updated slowly, essentially averaging over a long history of past outcomes. In contrast, a fixed value near one implies that expectations are updated quickly to match the most recent outcomes. Thus, the learning rate can be interpreted as the amount of influence each unpredicted outcome exerts on the subsequent expectation. These updated expectations can, in turn, be used to select actions, often through a soft-max function with an inverse-temperature parameter. This parameter can be adjusted to optimize the trade-off between exploiting actions known to be valuable in the present (emphasized at higher inverse temperatures) and exploring actions that might be valuable in the future (emphasized at lower inverse temperatures) [12], [13], [15], [25].

Recent work has highlighted the advantages of using learning rates that, instead of remaining fixed, are adjusted adaptively according to environmental dynamics [9]–[11], [26]–[28]. For example, adaptive learning rates can help ensure that expectations remain relatively stable in stationary environments but change rapidly in response to abrupt environmental changes. Consistent with this idea, human behavior in tasks containing abrupt changes conforms to models in which the influence of each outcome depends on the statistics of other recent outcomes. Such rational adjustments of learning rate are most prominent after changes in action-outcome contingencies that lead to surprisingly large prediction errors [9]–[11].

Here we consider in detail two of these change-point tasks. The first, an estimation task, requires subjects to predict the next in a series of outcomes (randomly generated numbers) [9]. Each outcome is drawn from a normal distribution with a fixed mean and variance. However, the mean of this distribution is occasionally reset at random times, producing abrupt change-points in the series of outcomes. Learning rates can be measured directly on a trial-by-trial basis, using predictions and outcomes plugged into Eq. 1. Previous work showed that subjects performing this task tended to use learning rates that were consistent with predictions from a reduced form of a Bayesian ideal-observer algorithm, including a positive relationship between error magnitude and learning rate. However, the details of this relationship varied considerably across individual subjects. Some subjects tended to use highly adaptive learning rates, including values near zero following small errors and values near one following surprisingly large prediction errors. In contrast, other subjects used a much narrower range of learning rates, choosing similar values over most conditions. This across-subject variability was described by a flexible model that could generate behaviors ranging from that of a fixed learning-rate delta rule to that of the reduced Bayesian algorithm, depending on the value of a learning rate “adaptiveness” parameter.

The second task is a four-alternative forced-choice task that includes occasional, unsignaled change-points in the probabilistic associations of monetary rewards for each choice target [11]. Learning rates are not measured directly, as in the estimation task, but rather inferred from model fits. The best-fitting models incorporate learning rates that increase transiently after unexpectedly large errors, although the magnitude of this increase differs across subjects. The existence of this kind of across-subject variability can have dramatic effects on the interpretation of best-fitting parameters from models that do not account for this variability explicitly. Here we illustrate this problem by fitting behavioral data corresponding to different forms of adaptive learning with delta-rule models that neglect adaptive learning entirely. However, we emphasize that this problem is not limited to adaptive learning but can also arise when neglecting other factors that can influence performance on learning tasks, such as a tendency to repeat choices [29], [30], and more generally whenever oversimplified models are fit to complex behavioral data.

We used simulations of the two tasks to illustrate how fitting models with fixed learning rates to behavior that is based on adaptive learning rates can lead to misleading conclusions. For each task, behavioral data were simulated using a delta-rule inference algorithm with different levels of learning-rate adaptiveness coupled with a soft-max function for action selection. These simulated data were then fit, using maximum-likelihood methods, with a simpler model that included two free parameters: a fixed learning rate and the inverse temperature of a soft-max action-selection process (see Text S1). In all cases, the simpler, fixed learning-rate model was preferred over a null model constituting random choice behavior, even after penalizing for additional complexity (e.g., using BIC or AIC; see Text S1). Despite passing these model-selection criteria, we highlight two misleading conclusions that might be drawn from these fits: biased estimates of adaptive learning and of exploratory behavior.

The problem of misestimating adaptive learning is depicted in Figure 1A & B. Panel A shows simulations based on the estimation task. For this task, learning rate is measured directly as the proportion of the current prediction error used to update from the current prediction to the next prediction [9]. As expected, variability in measured learning rates tended to increase with learning-rate adaptiveness. The average value of measured learning rates also tended to decrease with learning-rate adaptiveness, because change-points that dictate high values of adaptive learning rates were relatively rare in our simulated tasks (black circles and error bars reflect median and interquartile range, respectively, across 800 simulated trials).

**Figure 1:**
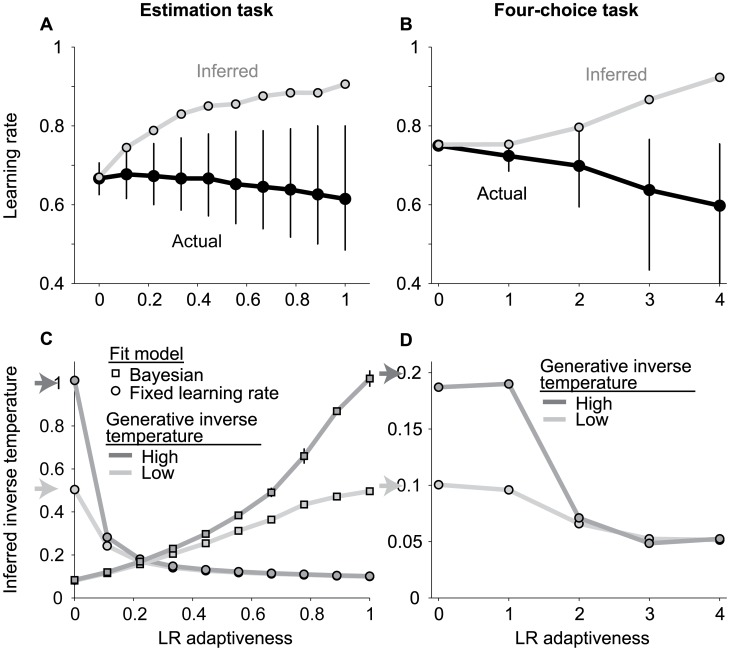
Learning-rate adaptiveness can be misinterpreted as elevated fixed learning rates and decreased inverse temperatures for the estimation (A,C) or four-alternative (B,D) tasks (see text). In all panels, the abscissa represents learning-rate adaptiveness (0 is equivalent to using a fixed learning rate; higher numbers indicate higher adaptiveness to unexpected errors). A & B. Actual (black) and model-inferred (gray) learning rates used by agents with different levels of learning-rate adaptiveness. Points and error bars represent the median and interquartile range, respectively, of data from six simulated sessions. C & D. Best-fitting values of the inverse-temperature parameter, intended to describe exploratory behavior, inferred using a fixed delta-rule (circles) or approximately Bayesian (squares) model. Shades of gray indicate the level of exploratory behavior of the simulated agent, as indicated. Arrows indicate the actual value of the inverse-temperature parameter used in the generative process. Points and error bars (obscured) represent the mean and standard error of the mean, respectively, of data from six simulated sessions.

The model fits, however, tell a different story. When behavior was simulated using a fixed learning rate (learning-rate adaptiveness = 0), the best-fitting models naturally captured the appropriate value. However, when behavior was simulated using increasingly adaptive learning rates, the fixed learning-rate models returned systematically larger estimates of learning rate than were actually used by the simulated subjects (Figure 1A, gray points).

Panel B shows simulations based on the four-choice task, for which we determined the learning rate on each trial based on its value in the internal, generative process used in the simulations. Data from this task tell a similar story. Simulated learning rates were lower but more variable for more adaptive models (black circles and error bars reflect median and interquartile range), yet fit learning rates were higher for these same models (Figure 1B, gray points). These data suggest that periods of rapid learning (i.e., following change-points) are more influential than periods of slow learning on maximum-likelihood fits of the fixed learning-rate parameter, which thus becomes biased upwards when the underlying learning rate is adaptive.

The problem of misestimating exploratory behavior is depicted in Figure 1C & D. We first simulated behavior on both the estimation task and the four-choice task using a fixed learning rate and an action-selection process governed by an inverse-temperature parameter. In each case, fits from a model with a fixed learning rate and an inverse-temperature process returned appropriate estimates of the inverse temperature used in the generative process (left-most circles in Figure 1C & D, corresponding to learning-rate adaptiveness = 0).

However, when the simulated subjects used increasingly adaptive learning rates, inverse-temperature fits from a fixed learning-rate model substantially overestimated the true variability in action selection (circles in Figure 1C & D: inferred inverse temperature decreases as learning-rate adaptiveness increases). Such biased parameter estimates were not simply a problem with the fixed learning-rate model. Fitting an alternative model that used optimal (maximally adaptive) learning rates [9], [31] to the behavior of the same simulated subjects yielded a complementary pattern of biases: the model accurately inferred the level of exploratory action selection for simulated subjects that choose learning rates adaptively but overestimated this quantity for subjects that used simpler strategies of less-adaptive, or even fixed, learning rates (squares in Figure 1C: inferred inverse temperature decreases as learning-rate adaptiveness decreases). For both models, these problems were not apparent from standard analyses of best-fitting parameter values, which had similar confidence intervals and covariance estimates for biased and unbiased fit conditions (see Text S1). These problems also did not simply reflect difficulties in estimating model parameters when the inverse temperature was low and behavior was more random, because the problem was also apparent when the inverse temperature was high. Thus, subtle differences in learning that were not accounted for by the inference model caused underestimation of the inverse-temperature parameter, which might be misinterpreted as increases in exploratory action selection.

Diagnosing these kinds of problems is difficult, especially when the subtle aspect of behavior that is missing from the model is unknown. Model-selection practices that compare likelihoods of various models (after either cross validation or penalization of parameter numbers) are useful for identifying the better of two or more models with respect to particular data sets. However, these practices require a priori knowledge of the models to be tested and cannot, by themselves, indicate what might be missing from the tested models. One might be tempted to interpret likelihoods directly and set a criterion for what might be considered a “good” model. However, these metrics cannot say whether or not a model is correct (or even sufficiently good, given that no fit model is truly correct). For example, consider a test of the suitability of a fixed learning-rate model for simulated subjects that can vary in terms of learning-rate adaptiveness and exploratory behavior. Similar values of AIC, BIC, and other likelihood-based quantities are obtained for fixed delta-rule models fit to two very different subjects: one who uses a fixed learning rate, which is consistent with the model, and relatively high exploration; and another who uses a highly adaptive learning rate, which is inconsistent with the model, and relatively low exploration. Interpretation of parameter fits from the latter case would be misleading, whereas parameter fits from the former would be asymptotically unbiased and thus more informative.

To overcome these limitations, it is sometimes effective to look for indications that a model is failing under specific sets of conditions for which behavior is heavily influenced by the assumptions of the model. For the case of adaptive learning, fixed learning-rate models fail to address adaptive responses to inferred change-points in the action-outcome contingency. Thus, it can be instructive to examine the likelihoods of these models computed for choice data collected shortly after change-points. For the case of the estimation task, a fixed learning-rate model shows an obvious inability to account for data from trials just after a change-point for all but the least adaptive simulated subjects (Figure 2A; dip in log-likelihood at trial 1). However, this approach is not effective for the four-choice task (Figure 2B).

**Figure 2:**
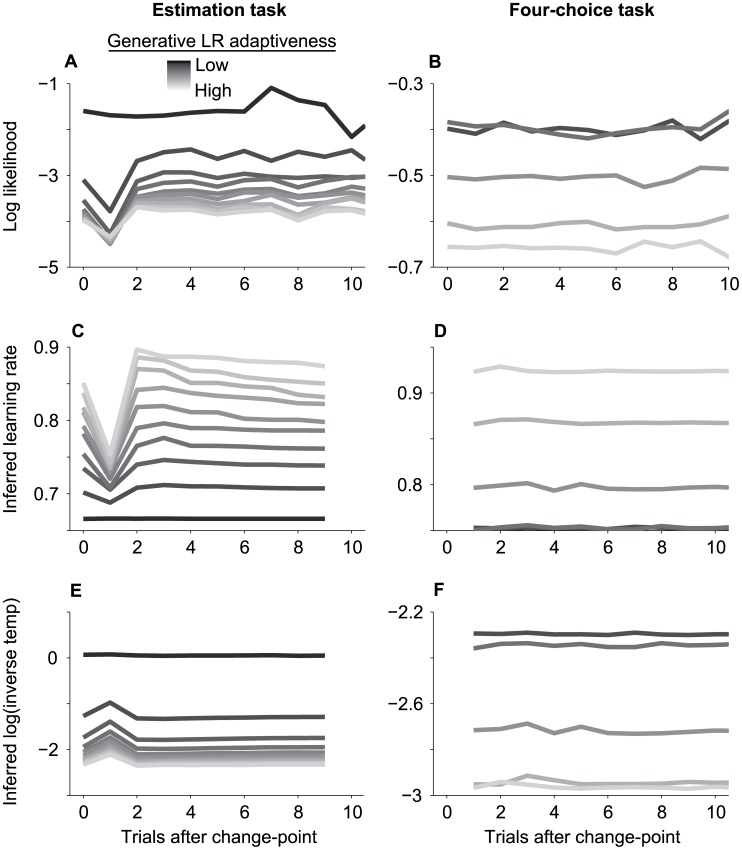
Poor fits from models that ignore learning-rate adaptiveness are easily identified in the estimation, but not the four-choice, task. A & B. Mean log-likelihood associated with a fixed learning-rate model, per simulated trial from the estimation (A) or four-choice (B) task, aligned to change-points in the generative process. Lighter shades of gray represent data from simulated agents with higher levels of learning-rate adaptiveness. C–F. Learning rates (C & D) or inverse temperatures (E & F) inferred from model fits that exclude log-likelihood information from trials occurring 0–10 trials after change-points (abscissa) for estimation (C & E) and four-choice (D & F) tasks. The transient changes in A, C, and E evident for all but the least adaptive simulated agents reflect the fixed learning-rate model's inability to account for behavior just following change-points on the estimation task; no comparable effects are evident for the four-choice task.

Another potentially useful approach for diagnosing misleading parameter fits is to compute these fits using subsets of data that might correspond to different best-fitting values of certain parameters. For the estimation task, eliminating data from trials immediately following change-points has dramatic effects on fits for both learning rate (Figure 2C) and inverse temperature (Figure 2E). However, this diagnostic approach is far less effective for the four-choice task, for which adjustments in learning rate occur with a longer and more variable time course following change-points (Figure 2D & F). Thus, for tasks like the estimation task that provide explicit information about the subject's underlying expectations, the insufficiency of the fixed learning-rate model can be fairly simple to diagnose. However, for tasks like the four-choice task in which information about the subject's expectations is limited to inferences based on less-informative choice behavior, parameter biases are still large (Figure 1B & D), but model insufficiency is far less apparent.

A sobering conclusion that can be drawn from these examples is that even when the parameter fits from a computational model are reasonably likely to produce a data set, and even when this likelihood is robust to perturbations in the specific trials that are fit or the settings of other parameters in the model, the model might still be missing specific features of the data. Missing even a fairly nuanced feature of the data (such as adaptive learning) can lead the parameters in the model to account for the feature in surprising ways. These unexpected influences can lead to parameter fits that, if interpreted naïvely, might suggest computational relationships that are unrelated to, or even opposite to, the true underlying relationships. Here we use an example from reinforcement learning, but the lessons apply to any model-fitting procedure that requires the interpretation of best-fitting parameter values. Certain parameters, like the inverse-temperature parameter in reinforcement-learning models, are particularly susceptible to this problem, because they are always sensitive to other sources of behavioral variability that are incompletely described by the rest of the model.

These challenges highlight the narrow wire on which the computational neuroscientist walks. On one hand, we seek to generalize a wide array of physiological and behavioral data from different tasks onto a tractable set of computational principles. On the other hand, the results that we obtain from each experiment are conditioned on assumptions from the particular model through which they are obtained. We believe that the goals of computational neuroscience are possible even in the face of this contradiction. Obtaining generalizable results depends on not only good modeling practices [32] but also the extensive use of descriptive statistics to dissect and interpret data from both experiments and simulated model data. For example, the estimation task described above was designed to allow learning rates from individual trials to be computed directly and not inferred via model fits to resulting choice behaviors. This approach revealed clear task-dependent effects on adaptive learning [9]. In principle, congruence between these kinds of direct analyses of behavioral data and fit model parameters can help support interpretations of those parameters and has the advantage of testing modeling assumptions and predictions explicitly rather than via comparisons of different model sets [8], [33], [34]. In contrast, inconsistencies between direct analyses and fit model parameters can help guide how the model can be modified or expanded—keeping in mind, of course, that adding to a model's complexity can improve its overall fit to the data but often by overfitting to specious features of the data and making it more difficult to interpret the contributions of individual parameters [35].

In summary, model fits to behavioral data can provide useful and important insights into the neurocomputational principles underlying such behavior but should not replace good experimental designs that explicitly isolate, manipulate, and/or measure the behavioral processes of interest. Combining such designs with both model fitting and other kinds of analyses can support steady progress in attaining a more general understanding of the neural basis for complex behaviors that is not overly tied to a particular model or behavioral test.

## Supporting Information

Text S1 Provides methods for simulations and model fitting as well as Bayesian information criterion values for each set of models.
(DOCX)
