# Supplementary material for: A Healthy Fear of the Unknown: Perspectives on the Interpretation of Parameter Fits from Computational Models in Neuroscience
Source: PLoS Comput Biol. 2013 Apr 4;9(4):e1003015. doi: 10.1371/journal.pcbi.1003015 (PMC3617224; doi:10.1371/journal.pcbi.1003015)
Supplement: Text S1 — Provides methods for simulations and model fitting as well as Bayesian information criterion values for each set of models. (DOCX) [file pcbi.1003015.s001.docx]

**Predictive-inference task simulations**

Task design*.* The subject’s task was to predict the value of each subsequent outcome presented in a sequence. Outcomes were generated as integer (rounded) picks from a normal distribution with a standard deviation equal to 35 (values between 5 and 40 gave similar results) and a mean that was initiated as a random value picked from a uniform distribution ranging from zero to 300. For each trial, a weighted coin flip determined whether the mean of the distribution would remain the same as on the previous trial (p=0.7, non-change-point trials) or whether the mean would be re-picked from a uniform distribution ranging from zero to 300 (p=0.3, change-point trial). Each sequence consisted of 800 outcomes.

Simulated behavior***.*** Task performance was simulated using the computational model that was best able to describe the range of behaviors of human subjects described previously [[1](#_ENREF_1)]. The model updates beliefs about the mean of the generative distribution after observing each new outcome according to the error made in predicting that outcome:

Eq. S1:


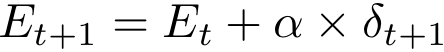


where *E* is the expected value of the distribution and δ is the difference between the actual outcome and the predicted one (*E*). The learning rate, α, is adjusted from trial-to-trial in accordance with estimates of uncertainty and change-point probability with a set of equations derived from the Bayesian ideal observer for the task. These inference equations for this model include two meta-parameters: hazard rate and learning-rate (LR) adaptiveness (previously termed likelihood weight) [[1](#_ENREF_1)]. Hazard rate controls the subjective expectation on the prior probability of a change-point, which in human subjects tends to overshoot the actual value and in our simulations was, accordingly, set to 0.5. LR adaptiveness determines the extent to which unlikely outcomes are used to recognize change-points and in turn adjust learning rates. A LR adaptiveness value of zero is equivalent to a fixed learning rate, whereas a LR adaptiveness value of one is consistent with optimal belief updating. To model the heterogeneity of human subjects in this regard to this parameter, we simulated ten subjects evenly spaced across the allowable range from zero to one.

We simulated behavior using the inference model, described above, in tandem with a probabilistic action-selection process using an inverse-temperature parameter. This process was implemented by computing the probability of choosing each option, *p*(*x*), according to a softmax function [[2](#_ENREF_2)]:


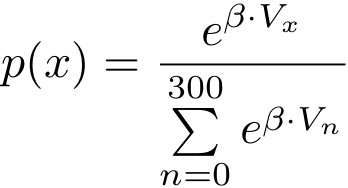


Eq. S2:

where *V_x_* is inversely proportional to the distance between the potential prediction (*x*) and the estimate derived from the inference model described above (*E_t_*), and β is the inverse temperature, which determines the variability in action selection and has previously been used as an indicator of exploratory behavior. Here we used inverse temperatures ranging from 0.2 to 1. For each set of parameters, the simulated subjects completed five task sessions.

Model fitting. We fit simulated behavior from the predictive-inference task using a fixed learning-rate model. This model updated beliefs according to Eq. S1, albeit with a fixed learning rate (α) for all trials from a given session. This model also used the same action-selection mechanism described above (Eq. S2). This model was fit to simulated behavior with learning rate and inverse temperature as free parameters, using a constrained search algorithm (fmincon implemented in Matlab) to minimize the negative log likelihood of the model relative to the simulated behavioral data.

**Four-alternative forced-choice simulations**

Task design*.* The four-alternative forced-choice task simulated here was previously developed and used by Krugel and colleagues [[3](#_ENREF_3)]. Subjects were asked to choose between four possible alternatives according to perceived value. After choosing an alternative, the subject was shown the value of the outcome associated with that choice. There were two possible outcome values: a high value (250 pts) and a low value (50 pts). For each trial, one (best) alternative is the most likely to yield a large reward. To maximize our ability to achieve reliable model fits, we simulated sessions with 10,000 trials in which outcomes were assigned to each possible choice by the following process:

1) A weighted coin flip determined whether the best target would remain in the same location as on the previous trial (the probability of a change = 0.1).

If Change: a new best target is sampled at random from all targets.

Otherwise: the best target remains in the same position as previously.

2) Outcome values were chosen at random (from the two possible values) for each alternative with *p*(high value) = 0.8 for the best alternative and 0.2 for all other alternatives.


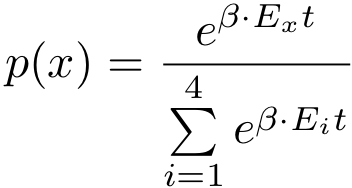
Simulated subject behavior*.* Behavior was simulated according to the adaptive learning-rate model used by Krugel and colleagues that was best capable of describing the behavior of human subjects [[3](#_ENREF_3)]. In brief, choices were selected according to a softmax action-selection rule that depended on a value function (q) and an inverse temperature term [[2](#_ENREF_2)]:

Eq. S3

After each trial, the value of the chosen option for the current timestep (E_I,t_) was updated according to the reward prediction error on that trial:


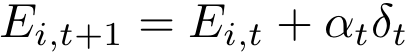
Eq. S4


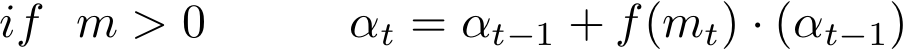
where δ reflects the difference between the actual outcome value, and α is the learning rate. The learning rate was adjusted on each trial according to the slope of the recent, unsigned prediction errors (*m*):


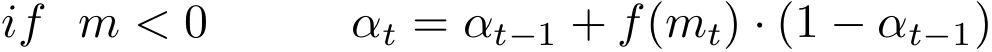
Eqs. S5


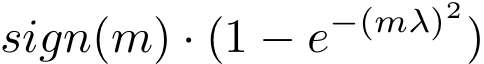
Thus, learning rate increased if the absolute value of the most recent prediction errors was large but decreased if the absolute value of recent prediction errors was small. The form of *f*(*m*) was a double-sigmoid transfer function:

Eq. S6

where the learning rate adaptiveness parameter, λ, determines the extent to which learning rates are altered according to recent absolute errors. When λ is equal to zero, learning rates become stable and thus maintain their initial value (which in our simulations was set to zero). When λ takes larger values, the learning rate becomes increasingly dependent on the slope of recent absolute prediction errors.

Model fitting. We fit simulated behavior with a model that included the same action-selection (Eq. S3) and learning mechanisms (Eq. S4) described above but used a fixed learning rate for all trials (instead of Eqs. S5 and S6). Thus, the model had two free parameters (learning rate and inverse temperature), which were fit simultaneously to simulated behavioral data by minimizing negative log likelihood using the Matlab function fmincon.

**Standard model-selection tests**

All model fits described in the main text were better descriptors of behavior than null models that reflected random choice behavior for the respective tasks, as measured by BIC or AIC (BIC values are shown in Figs. S1 and S2). That is, even the most ill-suited models (e.g., fixed learning-rate models fit to adaptive learning behavior) would not be rejected on the basis of appropriate likelihood-based tests.

**
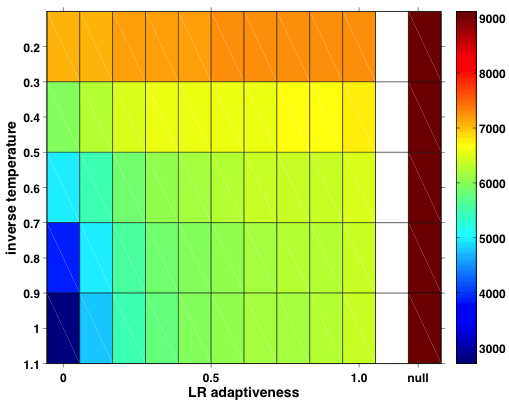
**

Figure S1: BIC values for all models fit to simulated behavioral data for the predictive-inference task. BIC values are represented in color (see legend to the right: hotter colors reflect higher BIC values; that is, worse fits) for each simulated value of inverse temperature and LR adaptiveness when fit using a fixed learning-rate model. For comparison, the BIC of a null model that reflects random choice activity is included (column on far right).**
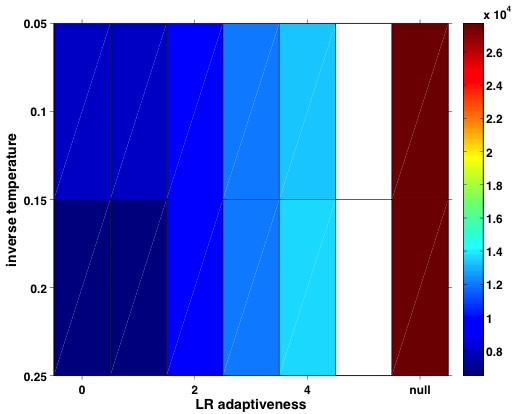
**

Figure S2: BIC values for all models fit to simulated behavioral data for the four-choice task. BIC values are represented in color (see legend to the right: hotter colors reflect higher BIC values; that is, worse fits) for each simulated value of inverse temperature and LR adaptiveness when fit using a fixed learning-rate rate model. For comparison, the BIC of a null model that reflects random choice activity is included (column on far right).**Parameter correlations**

In addition to extracting maximum likelihood parameter fits for each free parameter separately, we examined the covariance between fit values for the two parameters. First, we took the inverse of the Hessian matrix over negative log likelihood at the point in parameter space where this function was minimized (using fmincon in Matlab). This quantity is a standard estimator for the covariance of parameter estimates that can be derived from a single model fit [[4](#_ENREF_4)], and high covariance can be taken as an indication that a model is poorly conditioned [[2](#_ENREF_2)]. Although all of our simulations included non-zero (negative) covariance between the fit values of learning rate and inverse temperature, the covariance terms were no greater in magnitude for fixed-learning models fit to adaptive behavior (i.e., models that produce biased parameter fits) than then they were for fixed learning models fit to fixed learning behavior (i.e., models for which parameter fits were accurate).

**References**

1. Nassar MR, Wilson RC, Heasly B, Gold JI (2010) An approximately Bayesian delta-rule model explains the dynamics of belief updating in a changing environment. J Neurosci 30: 12366-12378.

2. Daw ND (2009) Trial-by-trial data analysis using computational models. In: Phelps E, Robbins T, Delgrado M, editors. Decision making, affect, and learning: Attention and performance XXIII. Oxford: Oxford University Press. pp. 3-38.

3. Krugel LK, Biele G, Mohr PNC, Li S-C, Heekeren HR (2009) Genetic variation in dopaminergic neuromodulation influences the ability to rapidly and flexibly adapt decisions. Proc Natl Acad Sci U S A 106: 17951-17956.

4. MacKay DJ (2003) Information theory, inference and learning algorithms. Cambridge: Cambridge University Press. 631 p.
